# TrkB overexpression in mice buffers against memory deficits and depression-like behavior but not all anxiety- and stress-related symptoms induced by developmental exposure to methylmercury

**DOI:** 10.3389/fnbeh.2014.00315

**Published:** 2014-09-12

**Authors:** Nina N. Karpova, Jesse Saku Olavi Lindholm, Natalia Kulesskaya, Natalia Onishchenko, Marie Vahter, Dina Popova, Sandra Ceccatelli, Eero Castrén

**Affiliations:** ^1^Neuroscience Center, University of HelsinkiHelsinki, Finland; ^2^Department of Neuroscience, Karolinska InstitutetStockholm, Sweden; ^3^Institute of Environmental Medicine, Karolinska InstitutetStockholm, Sweden

**Keywords:** perinatal methylmercury, anxiety, hippocampus, brain-derived neurotrophic factor, Bdnf transcripts, truncated TrkB, glyoxalase, glutathione reductase

## Abstract

Developmental exposure to low dose of methylmercury (MeHg) has a long-lasting effect on memory and attention deficits in humans, as well as cognitive performance, depression-like behavior and the hippocampal levels of the brain-derived neurotrophic factor (*Bdnf*)in mice. The Bdnf receptor TrkB is a key player of Bdnf signaling. Using transgenic animals, here we analyzed the effect of the full-length TrkB overexpression (TK+) on behavior impairments induced by perinatal MeHg. TK overexpression in the MeHg-exposed mice enhanced generalized anxiety and cue memory in the fear conditioning (FC) test. Early exposure to MeHg induced deficits in reversal spatial memory in the Morris water maze (MWM) test and depression-like behavior in the forced swim test (FST) in only wild-type (WT) mice but did not affect these parameters in TK+ mice. These changes were associated with TK+ effect on the increase in *Bdnf 2, 3, 4* and *6* transcription in the hippocampus as well as with interaction of TK+ and MeHg factors for *Bdnf 1, 9a* and truncated *TrkB.T1* transcripts in the prefrontal cortex. However, the MeHg-induced anxiety-like behavior in the elevated plus maze (EPM) and open field (OF) tests was ameliorated by TK+ background only in the OF test. Moreover, TK overexpression in the MeHg mice did not prevent significant stress-induced weight loss during the period of adaptation to individual housing in metabolic cages. These TK genotype-independent changes were primarily accompanied by the MeHg-induced hippocampal deficits in the activity-dependent *Bdnf 1, 4* and *9a* variants, *TrkB.T1*, and transcripts for important antioxidant enzymes glyoxalases *Glo1* and *Glo2* and glutathione reductase *Gsr*. Our data suggest a role of full-length TrkB in buffering against memory deficits and depression-like behavior in the MeHg mice but propose the involvement of additional pathways, such as the antioxidant system or TrkB.T1 signaling, in stress- or anxiety-related responses induced by developmental MeHg exposure.

## Introduction

Environmental risk factors (childhood abuse/neglect, exposure to environmental pollutants) drive pathological epigenetic reprogramming early in life which may trigger the development of mood disorders in adult humans and rodents (Weaver et al., 2004; Onishchenko et al., 2007, 2008; McGowan et al., 2009). Methylmercury (MeHg) is an environmental chemical contaminant of a major concern mainly for its detrimental effects on developing organisms that are more vulnerable to its toxicity (Ceccatelli et al., 2013). Therefore, advices for women and young children have been formulated in many countries. For instance, the U.S. Environmental Protection Agency reports that 2.3% of U.S. women 16–49 years of age have blood mercury concentrations higher than 5.8 micrograms per liter, which is the current reference dose (Birch et al., 2013). This means that approximately 1.4 million U.S. women at reproductive age have blood mercury concentrations posing a risk to their unborn children, which may result in 75,000 newborns per year with learning disabilities induced by *in utero* exposure to MeHg.

Perinatal exposure to low levels of MeHg impairs memory and attention in children of high fish-consuming populations and induces long-term impairments in cognitive performance in mice (Grandjean and Landrigan, 2006; Johansson et al., 2007; Onishchenko et al., 2007; Ceccatelli et al., 2013). We have previously shown that developmental MeHg-exposure induced long-lasting depression-like behavior and decreased hippocampal expression of the critical for brain functioning gene brain-derived neurotrophic factor (Bdnf), in adult mice (Onishchenko et al., 2008). Impaired Bdnf expression or signaling through its receptor TrkB was linked to learning and memory problems and to development of anxiety in several transgenic and stress models of brain disorders (Minichiello et al., 1999; Soliman et al., 2010; Karpova et al., 2011; Kemppainen et al., 2012; Lai et al., 2012). Developmental MeHg-exposure, however, did not alter the full-length *TrkB* transcript levels at least in the hippocampus (Onishchenko et al., 2008), and no other study linking the long-term effect of perinatal MeHg treatment to TrkB expression has been performed.

Several symptoms of neurodevelopmental disorders, including those in Rett syndrome and attention deficit hyperactivity disorder, may be improved with the antidepressant treatment (Maneeton et al., 2011; Gökben et al., 2012; Ghanizadeh et al., 2013). Chronic fluoxetine treatment in adulthood increased hippocampal *Bdnf* expression and reversed depression-like behavior in the MeHg-exposed mice (Onishchenko et al., 2008). Different antidepressant drugs activate TrkB receptor independently of Bdnf (Rantamäki et al., 2011) suggesting an antidepressant effect of increased TrkB activity. Moreover, genetic overexpression of the full-length TrkB receptor, TK, in postnatal neurons of the TK+ transgenic mice improved learning and reduce anxiety-like behavior in rodents (Koponen et al., 2004).

In the present study, using TK+ transgenic mice, we compared the influence of postnatal TrkB overexpression on behavioral alterations in adult mice with and without perinatal exposure to MeHg. Because developmental MeHg-induced changes in brain functioning may affect multiple behavioral domains, we used different behavioral tasks to investigate the long-term effect of perinatal MeHg-exposure on memory, anxiety- and depression-like parameters. In addition, since mood spectrum disorders in humans are linked to impaired circadian mechanisms (Etain et al., 2011), where Bdnf signaling plays an important role (Liang et al., 2000), we aimed at analyzing diurnal physiological parameters and behavior of the MeHg-exposed and TK+ mice using metabolic cages. We were interested to correlate behavioral data with the levels of *Bdnf* and *TrkB* transcripts in the prefrontal cortex and the hippocampus, the brain regions critical for learning/memory processes and development of mood disorders. Because MeHg-exposure might negatively impact on brain functioning through chronic induction of oxidative stress (Stringari et al., 2008; Gawryluk et al., 2011; Huang et al., 2011; Salim et al., 2011; Moretti et al., 2012), we also analyzed the expression of several antioxidant enzymes involved in scavenging of reactive oxygen species: glyoxalases 1 and 2 (*Glo1* and *Glo2*) and glutathione reductase *Gsr*. Our data show that TK overexpression primarily increased the levels of several *Bdnf* transcripts in the hippocampus and, in the MeHg mice, buffered against memory deficits and depression-like behavior. However, the stress-induced weight loss in the metabolic cages and some anxiety-related parameters were not ameliorated; these effects were associated with the MeHg-induced reduction in the hippocampal levels of the *Bdnf* transcripts *1* and *9a*, truncated *TrkB.T1* and the antioxidant enzymes.

## Material and methods

### Animals and MeHg treatment

All animal experiments followed the Council of Europe guidelines and were approved by the State Provincial Office of Southern and Eastern Finland. Overexpression of the full-length TrkB receptor (TK+) is targeted to postnatal neurons by the Thy-1 promoter (Koponen et al., 2004). TK+ mice on original BALB/c × DBA/2 background were backcrossed to more widely used C57BL/6J background for more than 10 generations and maintained as heterozygous in the Animal House, University of Helsinki. Heterozygous TK+ males were bred with C57BL/6J females (purchased from Harlan, Netherlands). Pregnant dams were housed individually starting from gestational day 6 and exposed to MeHg (CH_3_HgCl, Sigma-Aldrich, Finland) at the dose of 0.59 mg/kg/day (equivalent to 0.47 mg/kg/day of Hg described previously (Onishchenko et al., 2007) via drinking water *ad libitum* from gestational day 7 till day 7 after delivery. Concentration of MeHg in drinking water was adjusted based on body weight and water consumption which were checked daily. Pups were weaned at the age P21, and the male littermates were housed together (*N* = 3–5 per cage) in the Eurostandard cages (Tecniplast, Italy) Type II with floor area 370 cm^2^ for the 3-male litters or Type III with floor area 820 cm^2^ for the 4–5-male litters. Mice were kept under standard laboratory conditions (21°C, 12 h light-dark cycle, light at 6 AM) with free access to food and water. The wild-type (WT) and heterozygous TK+ littermates were used in the experiments.

### Measurement of total mercury content in brain tissue

Total mercury (Hg) content in the offspring forebrain tissue was measured in one pup from eight different litters (4 controls Ctrl and 4 MeHg) at the age P8. Hg measurements in the brain samples were carried out using the cold vapor atomic-absorption technique following alkaline digestion according to Magos (1971).

### Behavioral tests

The standard behavioral tests were performed by an experimenter unaware to the animal treatment group. Adult males (*N* = 9 Ctrl WT, 12 MeHg WT, 12 Ctrl TK+ and 7 MeHg TK+ mice) after 9 weeks of age were tested from 9 AM to 1 PM (with the exception for the comprehensive laboratory animal monitoring system (CLAMS) test) with the 3–4 day intervals in the order recommended for a multiple testing, starting with the tests for exploratory and emotional behavior followed by assessing learning/memory and finalized by the most stressful tests:

#### Elevated plus maze (EPM)

The maze consists of two open arms (30 × 5 cm), two closed arms (30 × 5 cm with 15 cm high non-transparent side- and end-walls) and a connecting central arena (5 × 5 cm). Test started from placing a mouse in the central arena facing to a close arm, and then the animal was allowed to explore the maze freely for 5 min in a dimly lit room. The animal was recorded with a video-tracking system (Noldus EthoVision XT 8.0, Noldus Information Technology, Wageningen, Netherlands) and the distance traveled, number of entries and the time spent in either area were calculated.

#### Open field (OF)

Testing was performed for 30 min in a well illuminated (300 lx) transparent acrylic cage (28.5 × 28.5 × 20 cm) (TSE, Bad Homburg, Germany). The cage was divided into two compartments: compartment near the walls (7 cm from walls) and central area compartment. Interruptions of infrared photo beams were used to calculate the distance travelled (cm) and time spent in compartments in 5-min intervals.

#### Fear conditioning (FC)

The fear memory is present as percentage of time spent freezing. Freezing behavior was measured with an automatic infrared beam detection system in the fear conditioning (FC) apparatus (TSE, Bad Homburg, Germany). Training was performed in a transparent Plexiglas chamber with metal grids on floor using two pairings of the conditioned stimulus, CS (total CS duration 30 s, 5 Hz, white noise, 80 dB), co-terminated with the unconditioned stimulus, US (1 s foot-shock 0.6 mA, inter-trial interval 30 s). The Baseline freezing to the context was measured for 2 min before the CS-US pairings. Contextual memory was tested 24 h later in the same chamber for 180 s. Unconditioned freezing to the Novel context (a black non-transparent Plexiglas chamber with planar floor) was measured 2 h later for 120 s, immediately followed by assessing a Cue memory with one CS presentation.

#### Morris water maze (MWM)

The system consisted of a black circular water tank conceptually divided into four quadrants of equal size, the distinctive 2-D distal cues around the tank and a computer-interfaced video-tracking system (Noldus EthoVision XT 8.0, Noldus Information Technology, Wageningen, Netherlands). The experiment consisted of the steps A–E. (A) The training with a hidden platform located at the annulus in one of the four quadrants was performed in twice daily sessions each consisting of three trials (60 s inter-trial interval) for 3 days (learning sessions 1–6). (B) The spatial memory of location of the trained platform was assessed 24 h later during the first probe trial without a platform (PT1, 60 s free swimming). (C) Next, the training continued with a platform located at the annulus in the opposite quadrant for 2 days (reversal learning sessions 7–10). (D) The spatial memory of the opposite platform’s location was assessed 24 h later during the second probe trial without a platform (PT2, 60 s free swimming). (E) The PT2 was followed by two control sessions of three trials with a platform and the position of the platform made visible by attaching yellow flag on top of it was changed for every trial. In every trial, the animal was released to swim in random positions facing the wall. The time to find a platform, swimming distance, velocity and thigmotaxis (swimming within 10 cm from the wall) were measured. In the probe trials PT1 and PT2, the spatial memory was estimated as the percentage of crossings of the target annulus compared to the other annuli.

#### Forced swim test (FST)

Test was conducted with a video-tracking system (Noldus EthoVision XT 8.0, Noldus Information Technology, Wageningen, Netherlands). Mouse was placed into a cylinder (diameter 18 cm, height 25 cm) filled with tap water at room temperature for 6 min. Frequency of immobility (passive floating) episodes, immobility time and latency to immobility were calculated.

#### Comprehensive laboratory animal monitoring system (CLAMS)

The CLAMS (Columbus Instruments, Columbus, OH) individually-housed metabolic cages (floor area 258 cm^2^) were used for automated, non-invasive and simultaneous monitoring of the following parameters: V O_2_ (volume of oxygen consumed, ml/h), V CO_2_ (volume of carbon dioxide produced, ml/h), energy expenditure (Kcal/h), accumulated food (grams) and activity counts over a 72-h period. The energy expenditure values were normalized to weight because of significant effects of the MeHg exposure and TK genotype on the animal weight. Animals were kept under standard laboratory conditions as described above.

### Molecular analysis

Animals were killed by carbon dioxide between 10 AM and 12 AM (when the behavioral testing normally took place) 3–4 days after the CLAMS test to ensure that there was no acute effect of the CLAMS on gene expression. The hippocampus and prefrontal cortex were dissected, immediately frozen on dry ice and kept at −80°C.

#### Preparation of the internal standard for real-time PCR quantification

Due to potential regulation of the housekeeping genes by the MeHg-exposure or TK+ genotype, the blasticidin resistance gene (*Blast*) was used for producing the internal standard, because no similar nucleotide sequence exists in the mouse genome. The *Blast* coding region was amplified from the plasmid pCoBlast DNA (Invitrogen, Carlsbad CA, USA) using the primers Blast-T7 and Blast-polyT listed in Table 1. The resulting PCR product, containing the T7 RNA polymerase promoter, *Blast* region and poly(A) tail, was purified from the agarose gel by QIAquick Gel Extraction kit (Qiagen, Valencia, CA, USA) and proceeded for *in vitro* transcription using T7 Transcription kit (Thermo Scientific, Finland). Poly(A) *Blast* RNA was purified with phenol/chloroform, extracted with ethanol, dissolved in nuclease-free water and stored at −80°C in small aliquots until use.

**Table 1:** **List of the primers used in the messenger RNA analysis**.

Gene	Forward primer	Reverse primer
*Bdnf1*	caagacacattaccttcctgcatct	accgaagtatgaaataaccatagtaag
*Bdnf2*	aagtgtttatcaccaggatctagccac	accgaagtatgaaataaccatagtaag
*Bdnf3*	ctttctatcatccctccccgagagt	accgaagtatgaaataaccatagtaag
*Bdnf4*	tgtttactttgacaagtagtgactgaa	accgaagtatgaaataaccatagtaag
*Bdnf6*	gaagcgtgacaacaatgtgactc	accgaagtatgaaataaccatagtaag
*Bdnf9a*	ggtctgaaattacaagcagatggg	accgaagtatgaaataaccatagtaag
*Bdnf total*	gaaggctgcaggggcatagacaaa	tacacaggaagtgtctatccttatg
*TrkB.FL*	gagctgctgaccaacctcca	gtccccgtgcttcatgtactca
*TrkB.T1*	taagatcccactggatgggtag	aagcagcacttcctgggata
*Glo1*	cctgatgacgggaaaatgaaag	gccgtcagggtcttgaatga
*Glo2*	ctctcagtgtcaaatgcctgtcaac	tcatagaacttcccacagccagcaac
*Gsr*	atgaagatggtttgtgccaaca	ccaatcccctgcatgtgaa
*Blast*	gggcatcttcactggtgtcaatgta	ctgttctcatttccgatcgcgac
*Blast-T7*	gataatacgactcactatagggcatcttcactggtgtcaatgtata	
*Blast-polyT*	gaaatcagctcttgttcggtcggtttttttttttttttttttt	

#### Messenger RNA analysis

Total RNA was extracted using QIAzol Lysis reagent (Qiagen Nordic, Sweden). 1 μg of total RNA was supplemented with 100 pg of poly(A) *Blast* RNA, treated with DNAse I (Thermo Scientific, Finland) and reverse transcribed using oligo(dT) primer and SuperScript III Reverse Transcriptase mix (Invitrogen, Carlsbad CA, USA). Each cDNA sample was amplified in triplicate with primers specified in Table 1 using Maxima SYBR Green real-time PCR mix (Thermo Scientific, Finland) and the Ct values were obtained using the Roche LightCycler 480 software. Relative quantification of template was performed using ΔΔCt method, with cDNA data being normalized to the *Blast* level. Control reactions with reverse-transcribed RNA without *Blast*, with only *Blast* RNA, and with RNA without reverse transcriptase were also performed. The data are present as % of Ctrl WT group.

### Statistical analysis

The values reported in the text, table and figures represent the means ± SEM. Data were analyzed using a factorial ANOVA or a repeated-measures ANOVA, taking the MeHg exposure and TK genotype as independent factors, followed by a *post hoc* Tukey test unless otherwise stated. A linear regression analysis was done using a Statview statistical package. A *P*-value < 0.05 was considered statistically significant.

## Results

### Effect of perinatal MeHg on the brain mercury levels and bodyweight

We accessed the whole-brain Hg levels in pups at postnatal day P8, the age when Hg accumulation is at highest levels in the brains of exposed offspring (Markowski et al., 1998; Newland and Reile, 1999). Similar to the previous data (Onishchenko et al., 2007, 2008), the exposure of pregnant dams to MeHg resulted in whole-brain Hg concentration of 1.18 ± 0.14 μg/g in their P8 pups (*n* = 5 pups) and in concentration below 0.0002 μg/g in the control (Ctrl) pups (*n* = 4 pups).

The MeHg exposure did not affect litter size: 7.6 ± 1 pups/MeHg litter and 8 ± 0.6 pups/Ctrl litter. The pups bodyweight averaged per litter was equal in both types of litters at the age P2 (MeHg 1.67 ± 0.15 g and Ctrl 1.54 ± 0.06 g), P8 (MeHg 4.80 ± 0.16 g and Ctrl 4.76 ± 0.25 g) and P21 (MeHg 8.49 ± 0.34 g and Ctrl 8.79 ± 0.14 g, *N* = 8 MeHg and 7 Ctrl litters, *P* > 0.05 for all comparisons, Student’s unpaired two-tailed *t*-test). There was no significant difference between the weights of males and females or WT and TK+ pups within each litter. In adult males, however, perinatal MeHg and TK overexpression had an additive effect on reduction in bodyweight (effect of the factors MeHg *F*_(1,36)_ = 18.43, *P* = 0.0001 and TK *F*_(1,36)_ = 12.59, *P* = 0.001) as measured before the onset of behavioral testing.

### Effect of MeHg exposure and TrkB overexpression on anxiety-like behavior

**Figure 1:**
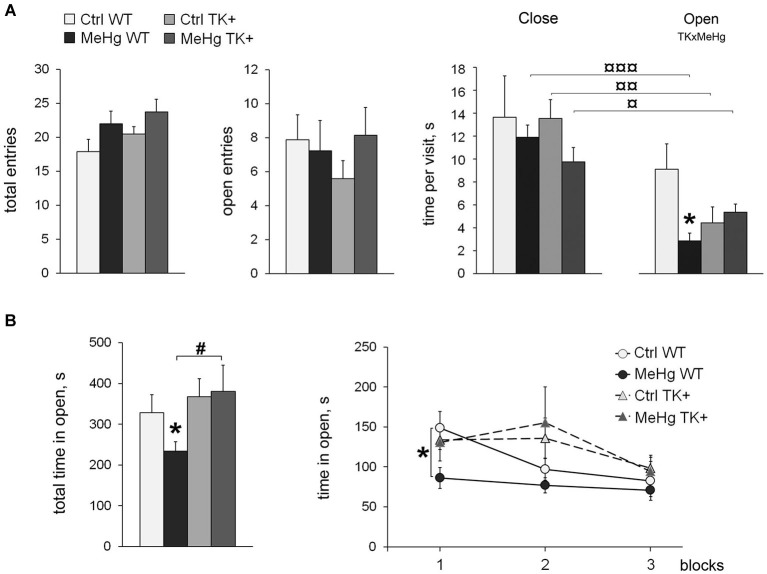
**Anxiety-like behavior. (A)** Elevated plus maze: the MeHg-exposed mice show increased locomotion, as well as reduced time per visit of the Open arms. All groups except Ctrl WT have decreased time per visit of the Open vs. Close arms; ^¤^ *P* < 0.05, ^¤¤^ *P* < 0.01, ^¤¤¤^ *P* < 0.001 paired two-tailed Student’s *t*-test. **(B)** Open field test: the time spent in open arena is shown as total and divided in three 10-min blocks. TK overexpression increases the time in open arena in the MeHg mice. Two-way ANOVA analysis: TK × MeHg significant interaction of the factors TK and MeHg. *Post hoc* Tukey’s group comparisons: * *P* < 0.05 MeHg vs. respective Ctrl group; ^#^ *P* < 0.05 TK+ vs. respective WT group. *N* = 9 Ctrl WT, 12 MeHg WT, 12 Ctrl TK+ and 7 MeHg TK+ mice.

Here we addressed the question how the MeHg exposure in early life affects anxiety-like behavior in adult WT and TK+ animals. Two behavioral tests for unconditioned anxiety were used: the elevated plus maze (EPM), and the open field (OF), tests. First, we detected the increased locomotor activity of the MeHg-exposed mice in the EPM test, with the significant main effects of the MeHg factor on the distance traveled (*F*_(1,36)_ = 5.01, *P* < 0.05) or total entries (*F*_(1,36)_ = 4.47, *P* < 0.05). There was no change in the number of entries to the open arms (Figure 1A). However, the averaged time that a mouse spent in the open arms during each visit (time per visit) was markedly reduced by the perinatal MeHg in the WT mice (interaction of the factors TK × MeHg *F*_(1,36)_ = 6.61, *P* < 0.05). Interestingly, all groups except for the Ctrl WT one showed a significantly shorter time per visit of the open arms as compared with the close arms (Figure 1A).

The OF test did not detect the increased locomotor activity in the MeHg animals. TrkB overexpression increased the total time spent in open (central lit) arena (TK effect *F*_(1,36)_ = 4.71, *P* < 0.05), while the MeHg exposure decreased it in only WT mice (Figure 1B). More specifically, the MeHg WT mice avoided the open arena during the whole 30 min-session, the Ctrl WT mice showed the decreased time in open in the second 10-min block, whereas both TK+ groups reduced exploration of the open arena only in the last 10-min block (repeated-measures ANOVA, interaction block × TK *F*_(2,72)_ = 3.21, *P* < 0.05; Figure 1B).

Taken together, the EPM and OF tests for unconditioned anxiety show that the perinatal MeHg exposure induced anxiety-like behavior in the adult WT mice, the effect that was lessened in their TK+ littermates.

### Effect of MeHg exposure and TrkB overexpression on cognitive abilities

**Figure 2:**
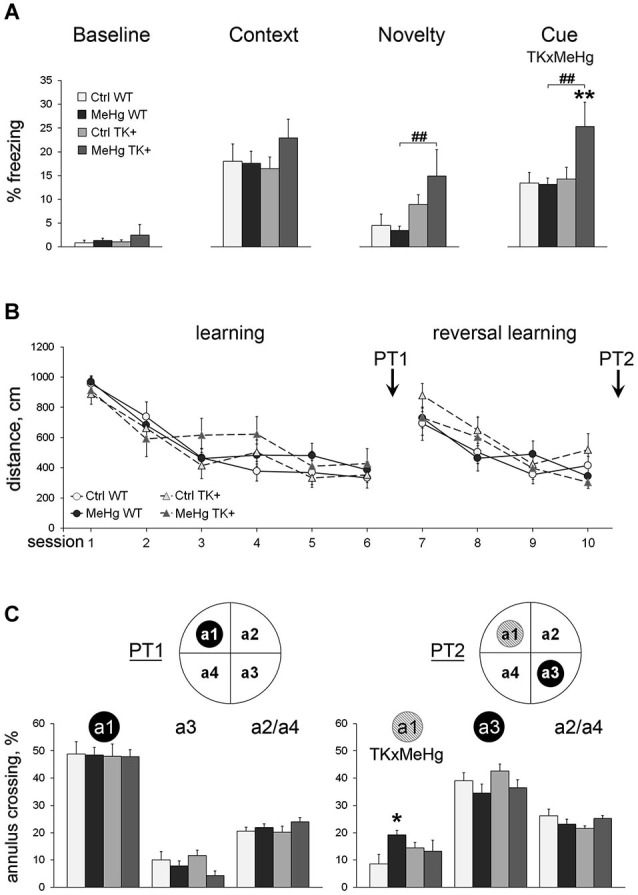
**Learning and memory. (A)** Fear conditioning: TrkB overexpression enhances generalized anxiety and conditioned cue memory in the MeHg mice. The freezing to the conditioning context was measured before training (Baseline) and next day after training (Context). The unconditioned freezing to the new context (Novelty) and one CS presentation (Cue) was measured after assessment of contextual memory. **(B)** Morris water maze: the MeHg mice show normal spatial learning throughout 10 sessions and **(C)** long-term memory during probe trials PT1 and PT2 but impaired reversal spatial memory during PT2. Inserts: schematic presentation of the MWM tank where the trained annuli are shown as the black circles, and the former trained annulus a1 during the PT2 trial is shown as a gray circle. a2/a4, the averaged percentage of crossing of the a2 and a4 annuli that have been never trained. Two-way ANOVA analysis: TK × MeHg significant interaction of the factors TK and MeHg. *Post hoc* Tukey’s group comparisons: * *P* < 0.05, ** *P* < 0.01 MeHg vs. respective Ctrl group; ^##^ *P* < 0.01 TK+ vs. respective WT group. *N* = 9 Ctrl WT, 12 MeHg WT, 12 Ctrl TK+ and 7 MeHg TK+ mice.

We first investigated if the MeHg exposure of the WT and TK+ mice had an effect on long-term fear memory. Using a FC paradigm, we found that neither MeHg treatment nor TK+ genotype affected manifestation of fear context memory 1 day after FC (Figure 2A). However, TrkB overexpression increased the freezing response to novel context (main effect of TK factor *F*_(1,36)_ = 9.20, *P* < 0.01; normalized to Baseline *F*_(1,36)_ = 10.64, *P* < 0.01) and cue presentation (main effect of TK factor *F*_(1,36)_ = 5.71, *P* < 0.05; normalized to Baseline *F*_(1,36)_ = 5.00, *P* < 0.05). This effect of TK+ genotype was manifested in only MeHg TK+ mice, which showed higher levels of fear generalization and conditioned auditory fear memory (Figure 2A), and resulted in significant interaction of the MeHg and TK factors during the cue presentation task (TK × MeHg *F*_(1,36)_ = 4.40, *P* < 0.05; normalized to Baseline *F*_(1,36)_ = 4.12, *P* < 0.05).

Next, we analyzed the MeHg and TK effects on spatial learning and relearning in a Morris water maze (MWM) test, a paradigm of learning and memory when animal should find the safe hidden platform using visual clues. The thigmotaxis was not affected by the MeHg or TK factors throughout the test (repeated ANOVA, *P* > 0.05 for the main effect of the factors or their interaction). The TK+ mice had increased velocity during learning to locate a hidden platform at the target annulus (repeated ANOVA, initial learning sessions 1–6: *F*_(1,36)_ = 6.57, *P* < 0.05; reversal learning sessions 7–10: *F*_(1,36)_ = 16.30, *P* < 0.001), but the distance swam to find a platform did not differ between the groups (Figure 2B). Thus, to specifically evaluate spatial memory in the probe trials PT1 (after initial learning) and PT2 (after reversal learning), when a platform was removed, we measured the percentage of crossing of the target annulus vs. the other annuli (Figure 2C). Spatial memory of the target annulus differed between the groups in neither PT1 (target annulus a1) nor PT2 (target annulus a3). But the MeHg exposure in only WT mice impaired extinction of the previously acquired memory of the former platform’s location (PT2 annulus a1; interaction TK × MeHg *F*_(1,36)_ = 5.24, *P* < 0.05).

### MeHg and TK+ effect on depression-like behavior

**Figure 3:**
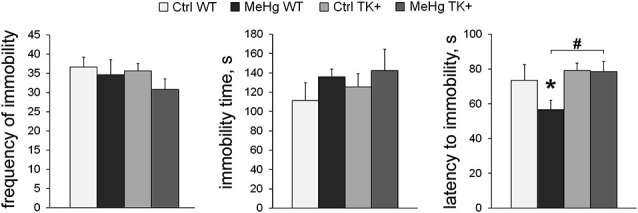
**Depression-like behavior**. In the forced swim test, TK overexpression increased the latency to immobility only in the MeHg mice. *Post hoc* Tukey’s group comparisons: * *P* < 0.05 MeHg vs. respective Ctrl group; ^#^ *P* < 0.05 TK+ vs. respective WT group. *N* = 9 Ctrl WT, 12 MeHg WT, 12 Ctrl TK+ and 7 MeHg TK+ mice.

We tested if TK overexpression may counteract with the effect of MeHg on depression-like parameters measured in the forced swim test (FST). Although the frequency of the immobility episodes and the total immobility time were not changed across the groups, the TK+ background prolonged the latency to immobility (TK effect *F*_(1,36)_ = 5.13, *P* < 0.05), and the MeHg exposure significantly shortened it in only WT mice (Figure 3). These data show that the depression-like effect of the perinatal MeHg exposure was not detected by the FST in the mice with genetically increased TrkB expression.

### Diurnal variation of metabolic parameters in the MeHg-exposed and TK+ mice

**Figure 4:**
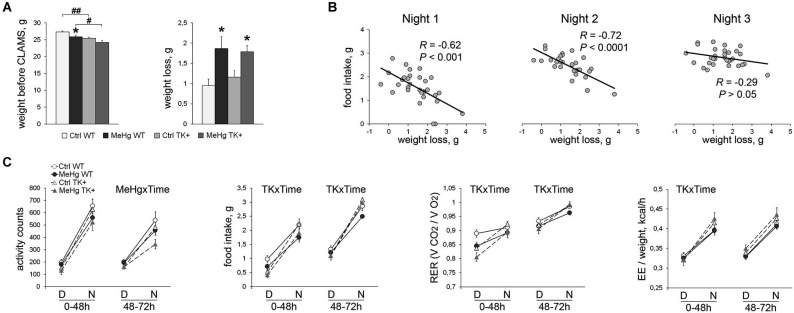
**Assessment of the diurnal and metabolic parameters for 72 h in CLAMS test. (A)** MeHg exposure resulted in significant weight loss in comparison with non-treated mice. *Post hoc* Tukey’s group comparisons: * *P* < 0.05 MeHg vs. respective Ctrl group; ^#^ *P* < 0.05, ^##^ *P* < 0.01 TK+ vs. respective WT group. **(B)** The total weight loss during the whole CLAMS test was negatively correlated with the food intake during the first and second active phases (Nights) suggesting a stressful period of adaptation to the CLAMS housing. *N* = 7 Ctrl WT, 10 MeHg WT, 8 Ctrl TK+ and 6 MeHg TK+ mice. **(C)** Diurnal variation in the activity and metabolic parameters during the adaptation 0–48 h and post-adaptation 48–72 h periods averaged per D (day inactive phase) or N (night active phase). RER, respiratory exchange ratio; EE, energy expenditure. Repeated two-way ANOVA analysis: TK × Time and MeHg × Time significant interactions of the factors TK or MeHg with Time. *N* = 8 Ctrl WT, 12 MeHg WT, 10 Ctrl TK+ and 7 MeHg TK+ mice.

To measure metabolic and diurnal parameters, the animals were weighted and then single-housed in the CLAMS metabolic cages over a 72-h period. The additive effect of perinatal MeHg and TK overexpression on decrease in bodyweight was preserved throughout the behavioral tests until the CLAMS testing (MeHg effect *F*_(1,33)_ = 9.07, *P* < 0.01; TK effect *F*_(1,33)_ = 15.48, *P* < 0.001; Figure 4A). Moreover, the MeHg-exposed mice lost significantly more weight in CLAMS (MeHg effect *F*_(1,33)_ = 10.16, *P* < 0.01; Figure 4A) suggesting that the adaptation to a new environment was more stressful for the MeHg than for the Ctrl mice. The metabolic CLAMS parameters are normally measured after a stressful adaptation period of a varying length, often up to 48 h (Liao et al., 2013). Because of significant negative correlation between the animal’s weight loss and the food intake during the first two nights (active dark phase) but not the last night of the CLAMS test (Figure 4B), we assumed that the 0–48 h period was stressful and analyzed it separately from the post-adaptation (48–72 h) period.

#### The 0–48 h stressful period (the left graph on each chart, Figure 4C)

The weight loss in the MeHg-mice was not the result of their increased activity, but was associated with decreased food intake (MeHg effect *F*_(1,27)_ = 4.75, *P* < 0.05) and respiratory exchange ratio (RER; MeHg effect *F*_(1,33)_ = 6.95, *P* < 0.05). The TK+ mice showed higher diurnal variation in food intake (TK × Time interaction *F*_(1,27)_ = 9.10, *P* < 0.01), RER (TK × Time *F*_(1,33)_ = 25.48, *P* < 0.0001) and energy expenditure (EE), or heat production, when normalized to the animal weight (TK × Time *F*_(1,33)_ = 20.05, *P* < 0.0001).

#### The 48–72 h post-adaptation period (the right graph on each chart, Figure 4C)

The TK+ mice again had higher diurnal variation in food intake (TK × Time interaction *F*_(1,27)_ = 11.47, *P* < 0.01) and RER (TK × Time *F*_(1,33)_ = 7.87, *P* < 0.01), but showed time-independent higher EE/weight values (TK effect *F*_(1,33)_ = 4.86, *P* < 0.05) compared to the WT mice. The MeHg-exposed mice had decreased nocturnal activity (MeHg × Time interaction *F*_(1,33)_ = 6.03, *P* < 0.05). Collectively, these data show that the decreased animal weight was associated with higher energy expenditure and diurnal variation of the metabolic parameters in the TK+ mice and higher stress sensitivity in the MeHg-exposed mice.

### Effect of perinatal MeHg on transcript-specific Bdnf and TrkB expression in the wild-type and TK+ mice

The statistical details of the MeHg and TK factorial effects on the expression of all analyzed transcripts are reported in Table 2.

**Table 2:** **Statistical analysis of gene expression**.

Gene	Treatment MeHg vs. Ctrl	Genotype TK+ vs. WT	TK × MeHg interaction
**Hippocampus**	***F*_(1,26)_; *P*-value**	***F*_(1,26)_; *P*-value**	***F*_(1,26)_; *P*-value**
*Bdnf1*	7.17; <0.05 ↓	ns	ns
*Bdnf2*	ns	16.96; <0.001 ↑	ns
*Bdnf3*	ns	12.48; <0.01 ↑	ns
*Bdnf4*	8.54; <0.01 ↓	17.46; <0.001 ↑	ns
*Bdnf6*	ns	9.81; <0.01 ↑	ns
*Bdnf9a*	4.45; <0.05 ↓	ns	ns
*Bdnf total*	ns	13.20; <0.01 ↑	ns
*TrkB.FL*	ns	198.31; <0.0001 ↑	ns
*TrkB.T1*	12.18; <0.01 ↓	ns	ns
*Glo1*	23.25; <0.0001 ↓	ns	ns
*Glo2*	5.59; <0.05 ↓	ns	ns
*Gsr*	4.72; <0.05 ↓	ns	ns
**Prefrontal cortex**	***F*_(1,32)_; *P*-value**	***F*_(1,32)_; *P*-value**	***F*_(1,32)_; *P*-value**
*Bdnf1*	ns	ns	11.82; <0.01
*Bdnf2*	5.17; <0.05 ↓	ns	ns
*Bdnf3*	4.47; <0.05 ↓	ns	ns
*Bdnf4*	ns	ns	ns
*Bdnf6*	ns	ns	ns
*Bdnf9a*	ns	ns	4.49; <0.05
*Bdnf total*	4.90; <0.05 ↓	ns	12.27; <0.01
*TrkB.FL*	ns	284.64; <0.0001 ↑	ns
*TrkB.T1*	ns	ns	4.71; <0.05
*Glo1*	ns	ns	ns
*Glo2*	ns	ns	ns
*Gsr*	ns	ns	ns

*The data were analyzed by the two-way ANOVA. Arrows indicate the expression levels that were increased (↑) or decreased (↓) by the factors MeHg and TK*.

**Figure 5:**
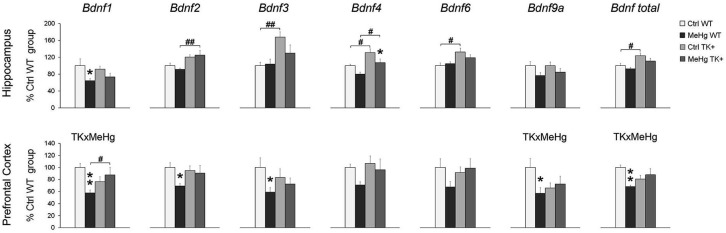
**Expression of the *Bdnf* transcripts**. The *Bdnf* expression was mainly decreased by MeHg and increased by TK+ background in the hippocampus, and dependent on factors’ interaction in the prefrontal cortex. Two-way ANOVA analysis: TK × MeHg significant interaction of the factors TK and MeHg. *Post hoc* Tukey’s group comparisons: * *P* < 0.05, ** *P* < 0.01 MeHg vs. respective Ctrl group; ^#^ *P* < 0.05, ^##^ *P* < 0.01 TK+ vs. respective WT group. *N* = 7–9 Ctrl WT, 8–11 MeHg WT, 9 Ctrl TK+ and 6–7 MeHg TK+ mice.

We determined the levels of several *Bdnf* transcripts because the expression of each of them is driven by a separate promoter (Pruunsild et al., 2011). In the hippocampus, the MeHg factor decreased the *Bdnf 1*, *4* and *9a* levels, while TK overexpression increased the *Bdnf 2*, *3*, *4*, *6* and total *Bdnf* transcript levels (Figure 5 and Table 2). In the prefrontal cortex, the MeHg exposure reduced the* Bdnf 1, 2, 3, 9a* and total *Bdnf* levels primarily in the WT mice, resulting in significant interaction of the TK and MeHg factors found for the *Bdnf 1*, *9a* and total *Bdnf* expression (Figure 5 and Table 2).

**Figure 6:**
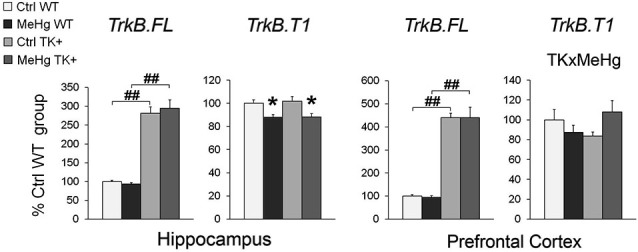
**Expression of the full-length *TrkB.FL* and truncated *TrkB.T1* transcripts**. MeHg exposure did not affect the *TrkB.FL* expression but reduced the *TrkB.T1* levels in the hippocampus and interacted with TK genotype for *TrkB.T1* in the prefrontal cortex. Two-way ANOVA analysis: TK × MeHg significant interaction of the factors TK and MeHg. *Post hoc* Tukey’s group comparisons: * *P* < 0.05 MeHg vs. respective Ctrl group; ^##^ *P* < 0.01 TK+ vs. respective WT group. *N* = 7–9 Ctrl WT, 8–11 MeHg WT, 9 Ctrl TK+ and 6–7 MeHg TK+ mice.

The developmental MeHg exposure did not affect the hippocampal and cortical levels of the TrkB full-length transcript *TrkB.FL* but down-regulated the major truncated TrkB isoform *TrkB.T1* in the hippocampus and, similar to the effect on *Bdnf* expression, significantly interacted with TK genotype for *TrkB.T1* expression in the prefrontal cortex (Figure 6 and Table 2).

### MeHg effect on the expression of the antioxidant enzymes

**Figure 7:**
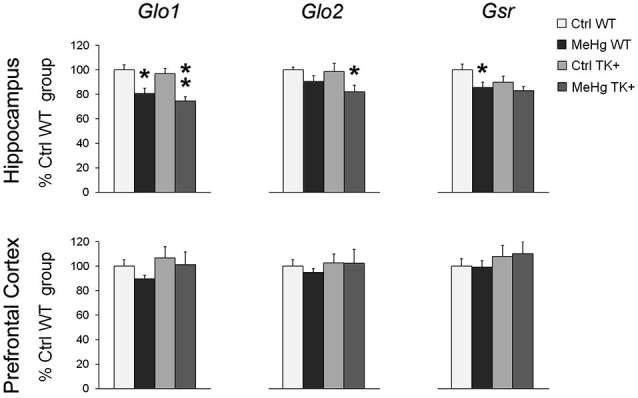
**Expression of the antioxidant enzymes**. MeHg exposure decreased the levels of *Glo1*, *Glo2* and *Gsr* in the hippocampus. *Post hoc* Tukey’s group comparisons: * *P* < 0.05, ** *P* < 0.01 MeHg vs. respective Ctrl group. *N* = 7–9 Ctrl WT, 8–11 MeHg WT, 9 Ctrl TK+ and 6–7 MeHg TK+ mice.

We found a significant MeHg-induced reduction in the hippocampal levels of *Glo1*, *Glo2* and *Gsr* transcripts coding for the important antioxidant enzymes (Figure 7 and Table 2). The TK overexpression did not affect these transcripts in either brain area.

## Discussion

Here we report for the first time that developmental MeHg exposure induces anxiety-related behavior in adult mice. These changes were detected in the classical tests for unconditioned anxiety and during the adaptation period in the CLAMS test, which can provide important data about stress-related physiological parameters. We found that the perinatal MeHg-exposure significantly reduced food intake and RER during adaptation to the novel CLAMS environment, resulting in substantial weight loss in both MeHg groups. Chronic MeHg exposure in adulthood was also reported to decrease weight gain in diabetic obese KK-Ay mice due to MeHg-induced toxicity in adipose tissue (Yamamoto et al., 2013). It is possible that MeHg toxicity in the brain induced sensitivity to stressful stimuli, provided either by behavioral manipulations or every-day home-cage activities, which could be among the mechanisms that mediate a long-term effect of perinatal MeHg on decreased bodyweight. Early-life stress is a main risk factor for development of psychopathologies in adulthood and is often associated with a hyperactive hypothalamic–pituitary–adrenal (HPA) axis (Heim and Nemeroff, 2001) and increased vulnerability to stress (Cirulli et al., 2009). Thus, it would be interesting to investigate the HPA axis reactivity and neuroendocrine responses to stress in early-life-MeHg exposed subjects.

The MeHg-induced behavioral changes were accompanied by the decreased levels of multiple *Bdnf* transcripts in the prefrontal cortex and hippocampus, and *TrkB.T1*, *Glo1*,* Glo2* and *Gsr* in the hippocampus. Bdnf gene structure with multiple promoters and different regulatory elements allows flexibility in *Bdnf* expression in response to various environmental stimuli. Among the *Bdnf* transcripts, *Bdnf1, Bdnf4* and *Bdnf9a* are the most responsive to neuronal activity *in vitro* (Pruunsild et al., 2011) and only these transcripts were down-regulated by the perinatal MeHg in the hippocampus. A key common mechanism for transcription driven by the corresponding *Bdnf* promoters p1, p4 and p9 is that they contain E-box-like elements which can be bound by the basic helix-loop-helix-PAS transcription factors Arnt2 and Npas4 in an activity-dependent manner (Pruunsild et al., 2011; Maya-Vetencourt, 2013). Since Npas4 plays an important role in the maturation of inhibitory synapses (Lin et al., 2008), perinatal MeHg could preferentially target the development of inhibitory system during hippocampal growth. In the prefrontal cortex, however, the MeHg effect was not restricted to the E-box-containing transcripts suggesting contribution of additional mechanisms in cortical *Bdnf* regulation. A possible mechanistic pathway for a brain area-specific MeHg effect could involve establishing of distinct DNA methylation marks at the *Bdnf* promoters during development (see also Onishchenko et al., 2008). A similar phenomenon has been recently identified for a *HSD11B2* gene methylation that differs between the hypothalamus and the cortex of prenatally stressed rats (Jensen Peña et al., 2012).

The effect of TrkB overexpression on the MeHg-induced *Bdnf* decrease was also brain area-specific: MeHg-independent in the hippocampus and MeHg-dependent in the prefrontal cortex. This effect may be due to activation of different tissue-specific or developmental pathways as discussed above, or to the higher *TrkB.FL* levels in the cortex (≈4.4 fold over WT levels) when compared to the hippocampus (≈2.9 fold over WT levels). Alternatively, a different neuronal/glia ratio between the brain regions (Herculano-Houzel, 2014) could account for the tissue-specific gene levels. Glia, including microglia and astrocytes, play a critical role in proper brain functioning, and was shown to be sensitive to oxidative stress and MeHg toxicity (Ni et al., 2012). Truncated TrkB.T1 is a major TrkB isoform in astrocytes, and its expression was shown to be reduced in the cortex of suicide completers via binding its 3′UTR by a microRNA Hsa-miR-185* (Maussion et al., 2012). The same group linked a deletion in TrkB promoter region, which could affect the levels of all *TrkB* transcripts including *TrkB.T1*, to childhood and adulthood anxiety in humans (Ernst et al., 2011). It could be suggested that the MeHg-induced decrease in the endogenous *TrkB*.*T1* expression that we observed in the hippocampus may impair Bdnf signaling in astrocytes, as well as reduce dendritic complexity in neurons and contribute to development of anxiety-like phenotype (Carim-Todd et al., 2009). Further studies are needed to investigate if the tissue-dependent gene expression observed in our study is due to neuronal or glial-specific transcription.

We detected a MeHg-induced decrease in the hippocampal levels of important antioxidant enzymes involved in methylglyoxal (*Glo1* and *Glo2)* and glutathione (*Gsr*) processing, suggesting a mechanism for a long-term MeHg effect on behavioral impairments. One study similarly reported the prenatal MeHg-induced reduction in cerebral glutathione peroxidase and reductase activities in mouse pups at the age P21, although adult mice were not investigated (Stringari et al., 2008). Chronic oxidative stress and accumulation of reactive-oxygen species has been consistently associated with anxiety- and depression-like behaviors, as well as cognitive dysfunctions in humans and rodents (Gawryluk et al., 2011; Salim et al., 2011; Moretti et al., 2012).

The TK overexpression did not show any beneficial effect on anxiety-like behavior in the EPM test in the adult MeHg animals, which can be explained by manifestation of anxiety phenotype in TK+ animals themselves. This result was unexpected considering a previous finding about the decreased EPM anxiety-like behavior in TK+ mice (Koponen et al., 2004). Differences in genetic background of TK+ mice in the present and previous studies may account for this discrepancy. Several behavioral studies report about more anxious phenotype of BALB/c and DBA/2 mice in comparison with C57BL/6J (Crawley et al., 1997; Hackler et al., 2006), which could explain the beneficial effect of TK overexpression on the EPM anxiety-like parameters found by Koponen et al. (2004). Our current EPM data are in line with reduced anxiety in *Ntrk2*-deficient heterozygote mice (Olsen et al., 2013) and with a critical role of TrkB activation in enhanced anxiety-like behavior in mice after status epilepticus (Liu et al., 2013).

In addition to the TK effect in the EPM test, we found that the TK+ background increased non-associative generalized fear responses (freezing to novel context) following FC, especially in the MeHg-exposed mice. Generalized hyper-responsiveness after an exposure to a traumatic event was suggested to model several features of post-traumatic stress disorder (PTSD; Balogh and Wehner, 2003), but could also model an adolescent-like increased emotionality found in late juvenile rodents with heightened fear generalization (Ito et al., 2009). Since PTSD-like phenotype is associated with learning and memory impairments, a second suggestion is more likely because the MeHg TK+ mice had better conditioned cue memory and did not show the defects in MWM reversal spatial memory. Further studies, which are focused on the analysis of fear renewal/reinstatement and spontaneous recovery using a FC-extinction paradigm, are required before drawing conclusion about potential PTSD-like phenotype of the MeHg TK+ mice.

The anxiety-related traits that we detected in the TK+ mice were not pronounced in less stressful OF test where TK+ background buffered the anxiety-like impairments of the MeHg mice. Moreover, although TK overexpression did not affect depression-like behavior in the Ctrl group, it prolonged the latency to immobility in the MeHg-exposed group. It is possible that TrkB overexpression leads to increased neuron number (Lähteinen et al., 2003) which, in our experiment, resulted in significant behavioral changes only in vulnerable to stress MeHg-treated animals. Consistently with the neurogenic hypothesis of depression (Eisch and Petrik, 2012), perinatal MeHg exposure reduces differentiation of neural stem cells and neurogenesis in adult dentate gyrus (Bose et al., 2012). At the same time, reduced TrkB expression is associated with increased apoptosis in depressed and suicide subjects (Dwivedi et al., 2009), and TrkB.FL deficiency in neuronal progenitors in adulthood impairs integration of newborn neurons into hippocampal neural circuits and consequently induces anxiety-like behavior in mice (Bergami et al., 2008). Thus, it could be hypothesized that TK overexpression specifically in the hippocampus, possibly via increased Bdnf expression observed in that brain area, supports more efficient integration of newly born neurons of MeHg mice into adult hippocampal network and improves some behavioral impairments. Additional studies using brain site-specific alteration of Bdnf signaling could help to verify this hypothesis. In early development, however, formation of functional neuronal networks by newborn neurons is active throughout the growing brain. During this sensitive period, emerging TK overexpression in transgenic animals could have stronger interference with Bdnf signaling throughout the brain of vulnerable to stress MeHg-exposed mice in comparison with the control ones, which could potentially lead to either positive or negative behavioral alterations in adulthood.

Depression- and anxiety-phenotypes are linked to impaired circadian mechanisms (Etain et al., 2011). Given the diurnal oscillation pattern of both Bdnf and TrkB (Bova et al., 1998) and their important role in sleep homeostasis (Faraguna et al., 2008) and circadian regulation (Liang et al., 2000), impaired Bdnf-TrkB signaling may promote development of mood disorders by disturbing the normal circadian cycle. In mice, chronic TrkB deficiency results in diminished circadian response to light (Allen et al., 2005). We found that the effect of TrkB overexpression on diurnal physiological parameters was opposite to that produced by TrkB-deficiency, but the TK+ background failed to ameliorate the diminished diurnal activity pattern in the MeHg-exposed mice that was also observed previously (Onishchenko et al., 2007). Strikingly, the increased diurnal pattern in food intake and a fuel selection parameter RER observed in the TK+ mice resemble those seen in calorie-restricted mice where these effects at least partially underlie their health and longevity (Bruss et al., 2010). The decreased weight of the TK+ mice without detectable changes in total food intake may as well be a result of increased EE contributing to the hypothesis of bodyweight regulation by Bdnf-TrkB signaling (Vanevski and Xu, 2013).

In summary, we can conclude that postnatal TrkB overexpression itself does not produce significant behavioral effects in C57Bl/6J mice, but it buffers against several behavioral impairments induced by perinatal MeHg. However, some parameters of anxiety-like behavior and associated decrease in the levels of activity-dependent *Bdnf* transcripts, *TrkB.T1* and the antioxidant enzymes in the hippocampus were permanently affected in adulthood by perinatal MeHg in a TK genotype-independent manner.

## Author contributions

Nina N. Karpova, Sandra Ceccatelli and Eero Castrén were responsible for the conception and design of the work. Nina N. Karpova, Jesse Saku Olavi Lindholm, Natalia Kulesskaya, Natalia Onishchenko, Marie Vahter and Dina Popova acquired and analyzed data. Nina N. Karpova, Natalia Kulesskaya, Sandra Ceccatelli and Eero Castrén interpreted data. Nina N. Karpova drafted the manuscript. All authors participated in critical revising and final approval of the manuscript, and agree to be accountable for all aspects of the work.

## Conflict of interest statement

The authors declare that the research was conducted in the absence of any commercial or financial relationships that could be construed as a potential conflict of interest.
